# Resting‐state functional connectivity modulates the BOLD activation induced by nucleus accumbens stimulation in the swine brain

**DOI:** 10.1002/brb3.1431

**Published:** 2019-11-07

**Authors:** Shinho Cho, Jan T. Hachmann, Irena Balzekas, Myung‐Ho In, Lindsey G. Andres‐Beck, Kendall H. Lee, Hoon‐Ki Min, Hang Joon Jo

**Affiliations:** ^1^ Department of Neurologic Surgery Mayo Clinic Rochester MN USA; ^2^ Center for Magnetic Resonance Research Department of Radiology University of Minnesota at Twin Cities Minneapolis MN USA; ^3^ Department of Neurologic Surgery Virginia Commonwealth University Health System Richmond VA USA; ^4^ Department of Neurology Mayo Clinic Rochester MN USA; ^5^ Department of Radiology Mayo Clinic Rochester MN USA; ^6^ Department of Biomedical Engineering and Physiology Mayo Clinic Rochester MN USA; ^7^ Department of Physiology College of Medicine Hanyang University Seoul South Korea

**Keywords:** blood‐oxygenation‐level‐dependent hemodynamic response, deep brain stimulation, functional connectivity change, functional magnetic resonance imaging, network effect, nucleus accumbens, resting‐state functional connectivity

## Abstract

**Introduction:**

While the clinical efficacy of deep brain stimulation (DBS) the treatment of motor‐related symptoms is well established, the mechanism of action of the resulting cognitive and behavioral effects has been elusive.

**Methods:**

By combining functional magnetic resonance imaging (fMRI) and DBS, we investigated the pattern of blood‐oxygenation‐level‐dependent (BOLD) signal changes induced by stimulating the nucleus accumbens in a large animal model.

**Results:**

We found that diffused BOLD activation across multiple functional networks, including the prefrontal, limbic, and thalamic regions during the stimulation, resulted in a significant change in inter‐regional functional connectivity. More importantly, the magnitude of the modulation was closely related to the strength of the inter‐regional resting‐state functional connectivity.

**Conclusions:**

Nucleus accumbens stimulation affects the functional activity in networks that underlie cognition and behavior. Our study provides an insight into the nature of the functional connectivity, which mediates activation effect via brain networks.

## Graphical abstract

Deep brain stimulation on nucleus accumbens of eight healthy swine evoked blood‐oxygenated‐level‐dependent (BOLD) activation in multiple cortical and subcortical brain regions.

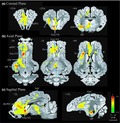

## 1 INTRODUCTION

While deep brain stimulation (DBS) is an established therapy for the treatment of essential tremors (Benabid et al., 1991), Parkinson's disease (PD) (Benabid, 2003; Group, 2001), and dystonia (Coubes et al., 2004), the recent use of DBS has expanded into the realm of neuropsychiatric disorders, that is, obsessive–compulsive disorder (OCD) (Greenberg et al., 2010, 2006; Mallet et al., 2008), treatment‐refractory depression (TRD) (Denys et al., 2010; Mayberg et al., 2005; Schlaepfer et al., 2008), addiction (Kuhn et al., 2009, 2007), and Tourette's syndrome (TS) (Houeto et al., 2005; Servello, Porta, Sassi, Brambilla, & Robertson, 2008). Cumulative results suggest that abnormal functional coupling between brain regions may be associated with neuropsychiatric diseases, that is, the cortico‐striato‐thalamo‐cortical (CSTC) circuit and the orbitofrontal cortex (OFC) (Greenberg et al., 2010, 2006; Rauch et al., 1994; Volkow, Fowler, Wang, Swanson, & Telang, 2007). However, the issue of how DBS alters functional coupling in potentially disease‐related brain networks and the nature of the biological mechanism that supports the DBS effect remain unclear (McIntyre & Hahn, 2010; Vitek, 2002).

NAc‐DBS has a demonstrated clinical efficacy, although the effects are serendipitous, and the mechanism of action is elusive, for alleviating the symptoms of comorbid depression and obsessive–compulsive mental symptoms (Bewernick et al., 2010; Hamani et al., 2014; Nuttin, Cosyns, Demeulemeester, Gybels, & Meyerson, 1999; Rasmussen, Greenberg, Mindus, Friehs, & Noren, 2000). Since DBS action is thought to alter functional coupling and regularize abnormal brain signals, previous studies have suggested that stimulating NAc influenced neural signaling between the ventral striatum and the prefrontal cortex (Figee et al., 2013), resulted in attenuating pathological hyperactivity (Baxter et al., 1992; McCracken & Grace, 2007; Rauch et al., 1994; Swedo, Rapoport, Leonard, Lenane, & Cheslow, 1989). However, it has recently been revealed that the DBS effect, while not specifically stimulating the NAc, appeared as widespread BOLD hemodynamic changes across the global brain, that is, throughout cognitive, limbic, and sensorimotor networks, as evidenced by functional imaging (Gibson et al., 2016; Knight et al., 2013; Krack, Hariz, Baunez, Guridi, & Obeso, 2010).

It is clear that NAc‐DBS induced its own BOLD activation pattern, as stimulating targets have their own unique, target‐specific modulation effects (Gibson et al., 2016; Knight et al., 2013; Krack et al., 2010; Min et al., 2012; Paek et al., 2015; Settell et al., 2017). However, it is unclear which specific neurobiological mechanism mediates broadly distributed BOLD activation patterns. Since BOLD activation can be found across anatomically heterogeneous regions that may not be directly connected to the stimulation site, an anatomical connection would not likely be the exclusive mechanism for delivering the DBS effect, albeit some anatomical connections have been found in DBS‐activated regions from diffusion tensor imaging (DTI) and fiber tracing studies in animal models (Britt et al., 2012; Leh, Ptito, Chakravarty, & Strafella, 2007; Lehéricy et al., 2004).

We investigated the pattern and extent of BOLD activation and changes in functional connectivity between activated brain regions after high frequency stimulation in a healthy pig model. In particular, we observed changes in inter‐regional functional connectivity (rsFC), focused on brain regions that evoked a significant BOLD response to NAc‐DBS, including executive, limbic, thalamic, and sensorimotor networks. Furthermore, we also conducted rsFC mapping (Biswal, Zerrin Yetkin, Haughton, & Hyde, 1995; Fox, Halko, Eldaief, & Pascual‐Leone, 2012) for each subject prior to the DBS implantation surgery in order to collect information on the relationship between the strength of rsFC and BOLD activation, as a potential action mechanism of DBS, which has been overlooked in previous DBS‐fMRI studies (Gibson et al., 2016; Min et al., 2012; Paek et al., 2015; Settell et al., 2017). The use of a large animal model in our study is presumably more representative of the human brain anatomy (Van Gompel et al., 2011) than small animal models (Albaugh et al., 2016), and therefore, our findings should be assumed to be general in nature, in terms of understanding the therapeutic effects of human DBS.

## 2 MATERIALS AND METHODS

### 2.1 Animal model and preparation

Eight male domestic pigs (*sus scrofa domesticus*, 8–12 months old, 25–30 kg) were initially sedated with an intramuscular injection of a ketamine (10–20 mg/kg) and xylazine (2.5 mg/kg) cocktail. Each animal was then orally intubated and mechanically ventilated by a medical‐grade pressure‐driven mechanical ventilator (respiration cycle: 12 per min) with a 7:3 N_2_O:O_2_ medical gas mixture. Anesthesia was maintained with a constant gas flow of isoflurane (concentration: 1.2%–1.4% for imaging experiment and 2% for DBS surgery). Animal physiology was measured with a MR compatible pulse oximetry and capnography sensor (Nonin Medical Inc) and maintained at normal conditions (heart rate: ~120 bpm, SpO_2_: 98%–100%, and end‐tidal CO_2_: 3.5%–4%). The rectal temperature (PhysiTemp Instrument) was monitored (37 ± 1°C) and maintained using a heated circulating water blanket. Animal surgical procedures and experimental protocol were approved by the Institutional Animal Care and Use Committee of the Mayo Clinic.

### 2.2 Experimental outline

**Figure 1:**
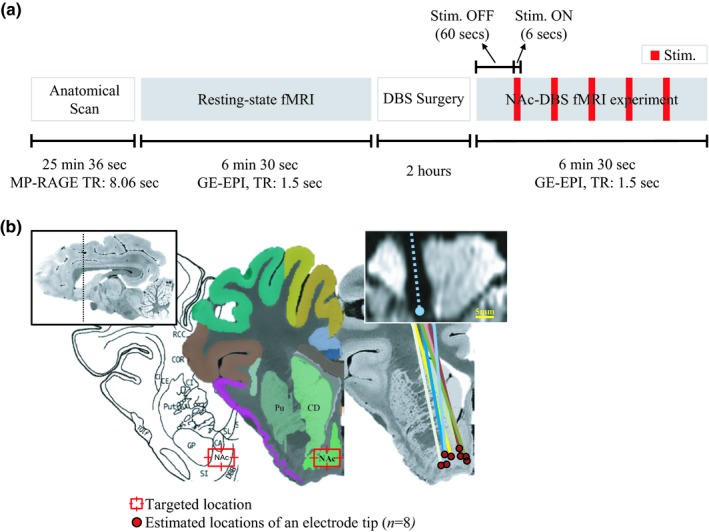
(a) Schematic diagram depicting the experimental procedure and (b) the placement of electrode in the placement of subjects on the pig brain atlas (Saikali et al., 2010). The red dots and colored lines indicate the estimated position of an individual electrode tip and body, respectively (*n* = 8). The postsurgical echo‐planar image in a coronal view is shown on the top right (a single subject), wherein the signal dropout induced by electrode body. See for the abbreviations in Appendix A

Figure 1a outlines the experimental timeline. After initial sedation and intubation, the animal was delivered to a scanner for imaging for an individual anatomical scan (25 min 36 s). The anatomical scan was followed by a resting‐state functional MRI (6 min 30 s). After resting‐state functional imaging, electrode implantation surgery was followed (2 hr) and the DBS‐fMRI experiment was carried out (6 min 30 s).

#### 2.2.1 Anatomical imaging

High‐resolution anatomical T1‐weighted images were obtained with a GE 3‐Tesla Signa Excite scanner using a custom‐built 8‐channel surface‐type radio frequency (RF) coil with following imaging protocols: A 3D magnetization prepared radio frequency pulses and rapid gradient echo (MP‐RAGE) sequence, TR/TE = 8.06/3.3 ms, inversion time = 1,000 ms, flip angle = 8°, slice thickness = 0.8 mm, matrix size = 300 × 300 × 108, field of view (FOV) = 240 × 240 × 87 mm^3^, average number = 2, and total scan time = 25 min 36 s.

#### 2.2.2 Resting‐state functional imaging

After anatomical imaging, resting‐state functional imaging was conducted with the following imaging protocols: gradient echo echo‐planar imaging (GE‐EPI), TR/TE = 1,500/40 ms, slice thickness = 2.4 mm, 19 slices, field of view (FOV) = 160 × 160 mm^2^, matrix size = 96 × 96, voxel resolution = 1.67 × 1.67 × 2.4 mm^3^, and total scan time = 6 min 30 s.

#### 2.2.3 DBS electrode implantation

A quadripolar DBS electrode (Model 3389; Medtronic Inc.) was implanted targeting to NAc of the left hemisphere (Knight et al., 2013; Min et al., 2012). The coordinates (arc, collar, and depth) on a stereotactic frame (Leksell; Elekta Co) were determined by COMPASS planning software (COMPASS International Innovations) based on NAc location identified from individual subject's anatomical brain images (Knight et al., 2013) (Figure 1). Microdrive (Alpha Omega Co.) was used to guide the implantation of a DBS lead. All of these systems can minimize the displacement of an electrode from a targeted position (<1 mm), as shown in our previous phantom tests (Min et al., 2014).

#### 2.2.4 DBS stimulation parameters and DBS‐fMRI acquisition

Following DBS surgery, each animal underwent functional imaging with simultaneous NAc stimulation. Each stimulation block consisted of a 6‐s stimulation train followed by a 60‐s off period stimulation (Figure 1a). The block was repeated five times per scan with the initial baseline period. The total time per scan was 6 min and 30 s. The imaging parameters were the same with those in the resting‐state scan were used.

The stimulation parameters were as follows: biphasic and bipolar pulse, voltage = 5 V, pulse frequency = 130 Hz, and pulse duration = 100 μs. These parameters were selected based on results from our previous study wherein we found a robust and reproducible BOLD activation in multiple trials across different subjects (Knight et al., 2013; Min et al., 2012; Paek et al., 2015; Settell et al., 2017).

### 2.3 fMRI data preprocessing

Resting‐state and DBS‐fMRI data were processed using the AFNI software (Cox, 1996). Animal physiological data were collected to remove image artifacts. Respiration cycle and cardiac pulsations were measured by a respiratory bellows positioned at the level of the abdomen and a pulse oximetry placed on the animal's left ankle. Data were recorded though the scanner (3T Signa Excite MRI scanner; GE Medical Systems), and timing was synchronized with scan start and stop of EPI sequence. To remove physiological artifacts, regressors for modeling respiration and cardiac activity, and respiration volume per time (RVT) were created and subtracted out from individual BOLD time course in slice by slice, according to the pipe line of the RETROICOR + RVT (Birn, Diamond, Smith, & Bandettini, 2006).

Spike removal, slice‐timing correction, and within‐subject motion correction with six parameters (three translations and three rotations of *x*‐, *y*‐, and *z*‐axis) were applied. Individual subject's anatomical and functional images were coregistered to the pig brain atlas using a 9‐parameter linear registration (3‐translation, 3‐rotation, and 3‐scaling) with the cost function of the Hellinger distance (Mallet et al., 2008), and the alignment of coregistration was then visually checked each time. Prior to statistical analysis, spatial smoothing was applied using 3‐mm full‐width‐half‐maximum (FWHM) isotropic Gaussian kernel. A temporal filter was not used in this study, because the physiological artifacts were regressed out, and temporal filtering could influence the temporal synchronization between imaging and stimulation onset (Davey, Grayden, Egan, & Johnston, 2013).

### 2.4 DBS‐fMRI BOLD activation map

The general linear model (GLM) analysis was adopted to detect stimulation‐induced BOLD activation and measure the amplitude of signal chance. An individual analysis was carried out first, and a group‐level (*n* = 8) statistical analysis was then performed (3dDeconvole and 3dttest, AFNI). In the group‐level analysis, significant BOLD activation was detected with the threshold, set as *p* < .05 (*n* = 8, *t* > 3.49, false discovery rate [FDR] corrected). The coordinates, *t*‐scores, and percent signal changes were summarized. The activation map was created with different color encoding based on the percent change in a signal and overlaid on the pig brain atlas in three view planes (axial, coronal, and sagittal).

### 2.5 Region of interest generation

**Figure 2:**
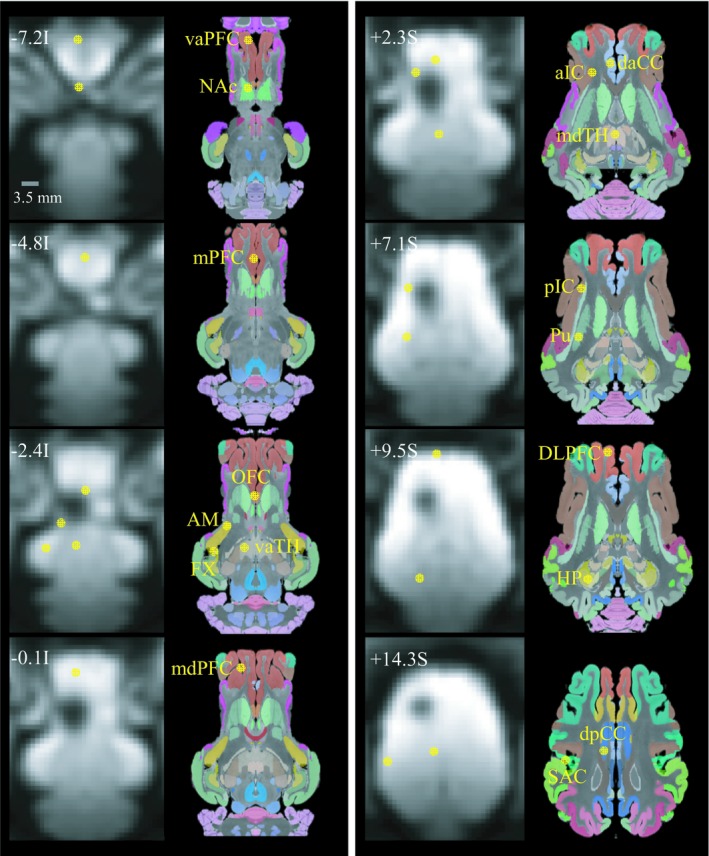
Region of interests (ROIs). The location and perimeter of region of interests (ROIs) are shown as yellow circles on postsurgical EPI images. Each region consisted of voxels in a radius within 1.7 mm from the location of the peak BOLD activation. The anatomical labels were presented together for reference. See for the abbreviations in Appendix A

Figure 2 shows the locations of the region of interests (ROIs) used in this study (see Appendix A for list of ROI abbreviations). The location of ROIs was determined based on a group‐level BOLD activation map, wherein significant stimulation‐induced activation was observed.

BOLD time courses for each ROI were generated. We first created a sphere mask (radius = 1.7 mm) and applied it on the center location of each ROI. We then extracted all time courses within the mask and averaged them individually, and generated the group‐averaged ROI time courses (*n* = 8).

### 2.6 Functional connectivity analysis

Functional connectivity (FC) analyses were conducted on data obtained during resting, stimulation, and poststimulation state. To calculate the resting‐state FC, the Pearson correlation coefficient (CC) was calculated between ROI time courses. For estimating stimulation and poststimulation‐state FC calculations, individual time courses were divided into two time periods: the first 30‐s data points after stimulation onset (stimulation state FC) and the later 30‐s data until the beginning of the following stimulation block (poststimulation state FC). For each subject, coefficient matrices were averaged across blocks, generating a single stimulation‐ or poststimulation‐state matrix. Finally, individual subject's CC matrices were normalized by Fisher's Z‐transformation and averaged into a group‐level CC matrix (*n* = 8). To detect significant FC during resting state, one‐sample *t* test was applied. To detect the significant change of FC, two‐sample *t* test was used between resting‐ and poststimulation‐state CC matrix.

### 2.7 The relationship between resting‐state functional connectivity and the BOLD response

Pearson's CC (*r*) and slope of linear regression (*s*) were estimated between the resting‐state functional connectivity (rsFC) of a given ROI to the NAc and the BOLD response of the ROI. In the voxel‐wise analysis, the same calculation was conducted: the sphere mask (radius = 1.7 mm) was applied to individual voxel in functional networks, and a voxel‐wise averaged time course was obtained. Then, statistics (*r* and *s*) between rsFC‐to‐NAc and BOLD response of a given voxel were estimated. Data points were categorized into five functional networks to plot separately.

The correlation and regression analyses were also applied between inter‐ROI rsFC and inter‐ROI FC change in poststimulation state. The same sort of analysis was applied to analyze the relationship between co‐activation of ROIs and inter‐ROI FC change in poststimulation state. In those analyses, the absolute CC value was considered.

### 2.8 Postsurgical evaluation of DBS electrode placement

The placement of the lead and location of electrode tip was examined by reconstructing an electrode‐induced image artifact on individual EPI image volumes (Horn & Kühn, 2015). Intensity thresholding was applied to the axial plane of a single EPI image slice. The signal dropout region induced by the image artifact was isolated, and centroids of the region were marked along with the slice direction. By interpolating those marks, the lead placement could be reconstructed on individual anatomical image volumes. The location of the electrode tip with contacts 0 and 1 was then estimated and mapped on the pig brain atlas (Figure 1b).

### 2.9 Comparison of EPI signal intensity in stimulation on and off periods

Echo‐planar imaging image volumes, obtained during stimulation on and off periods, were averaged across blocks. Voxel‐wise signal intensity was extracted within and adjacent area of the signal dropout region. Data points of stimulation on and off period were plotted separately with spatial distance. The statistical analysis (two‐sample *t* test) between the signal intensities of stimulation on and off blocks was conducted to detect the significance of signal difference in conditions.

## 3 RESULTS

### 3.1 BOLD activation induced by NAc‐DBS

**Figure 3:**
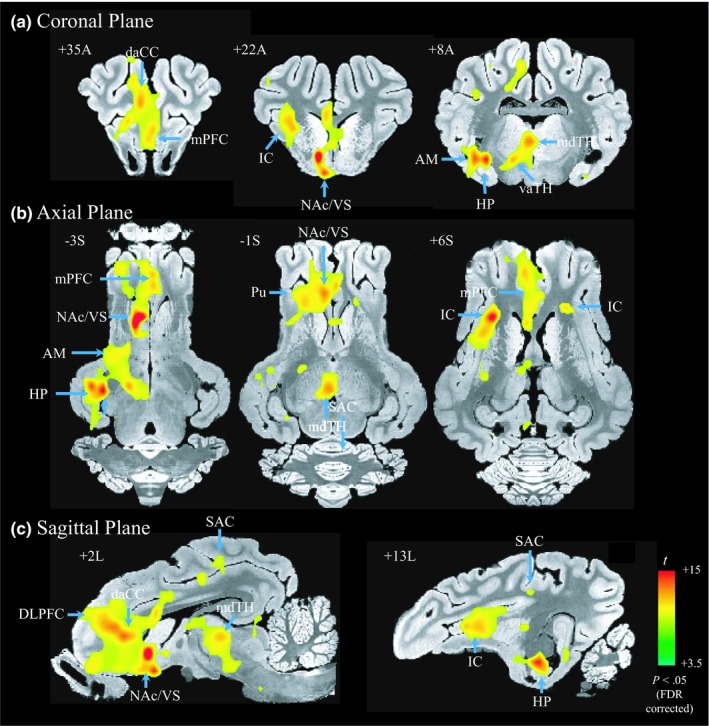
Group‐level BOLD activation map of a 130 hz NAc‐DBS (*n* = 8; *t* > 3.5; *p* < .05, false discovery rate [FDR] corrected). Activated regions are shown in (a) coronal, (b) axial, and (c) sagittal views on the pig brain atlas (Saikali et al., 2010), and the *t*‐scores for activation are denoted by colors. NAc‐DBS induced BOLD activation in multiple cortical and subcortical brain regions. Note that DBS electrodes were implanted in the left hemisphere for all subjects. For abbreviations, see Appendix A

NAc‐DBS evoked significant BOLD activation in multiple cortical and subcortical brain regions, but was limited to the ipsilateral hemisphere (Figure 3). The highest amplitude of evoked BOLD activation was found in the ipsilateral NAc (signal change: 1.27 ± 0.19%, beta coefficient: 1.6, *t* = 12.11, *p* < .05, false discovery rate [FDR] corrected), indicating the current spread near the DBS electrode tip likely evoked a direct neuromodulatory effect (McIntyre, Mori, Sherman, Thakor, & Vitek, 2004). However, it must be noted that BOLD activation was also evident beyond the stimulation locus, that is, prefrontal, cingulate, insular, and sensorimotor cortices (*p* < .05, FDR corrected) (see Table 1 for the summary of activated clusters). Thus, the results show NAc‐DBS induced both local and distal modulatory effects across multiple functional networks including executive, limbic, thalamic, and sensorimotor networks. Interestingly, such effect was primarily lateralized to the left hemisphere, ipsilateral to the DBS electrode (Figure 3b). Only a few regions in the contralateral hemisphere (pIC, CD, Pu, and premotor cortex) showed significant BOLD activation (*p* < .05, FDR corrected), demonstrating that the DBS effect may be unilateral.

**Table 1:** The summary of BOLD activation clusters

Regions	MNI coordinate [mm]			*t*	GLM Beta	Max % change (±1 *SEM*)
	*X*	*Y*	*Z*			
Ipsilateral stimulation hemisphere (left)						
Executive/prefrontal						
Dorsal lateral prefrontal cortex (DLPFC)	5.10	−35.27	10.45	5.61	1.37	0.76 ± 0.20
Medial prefrontal cortex (mPFC)	0.90	−34.97	−4.85	7.66	1.14	0.79 ± 0.14
Medial dorsal prefrontal cortex (mdPFC)	5.10	−35.27	0.25	7.40	1.60	1.06 ± 0.21
Orbitofrontal cortex (OFC)	0.90	−23.27	−2.45	6.81	0.91	0.79 ± 0.42
Ventral anterior prefrontal cortex (vaPFC)	2.40	−38.27	−6.95	6.36	1.05	0.68 ± 0.15
Executive/insular						
Anterior insular cortex (aIC)	7.20	−26.58	2.05	7.77	1.45	1.05 ± 0.14
Posterior insular cortex (pIC)	10.80	−23.27	7.15	13.64	1.20	1.01 ± 0.14
Limbic/basal ganglia						
Amygdala (AM)	10.80	−12.78	−2.45	6.58	1.87	1.29 ± 0.15
Fornix (FX)	15.90	−4.38	−2.45	11.78	0.59	0.54 ± 0.12
Nucleus accumbens (NAc)	2.40	−21.47	−7.25	12.11	1.63	1.27 ± 0.19
Putamen (Pu)	12.60	−6.48	6.55	4.99	0.47	0.44 ± 0.12
Hippocampus (HP)	10.90	7.50	9.60	5.90	0.31	0.36 ± 0.12
Dorsal anterior cingulate cortex (daCC)	2.40	−29.88	2.35	10.20	1.43	1.06 ± 0.18
Dorsal posterior cingulate cortex (dpCC)	4.20	−6.48	14.65	7.61	0.23	0.27 ± 0.12
Sensorimotor						
Somatosensory association cortex (SAC)	15.30	−0.48	21.25	4.75	0.54	0.68 ± 0.08
Thalamus						
Medial dorsal thalamus (mdTH)	0.60	−4.07	2.35	9.73	0.84	0.83 ± 0.12
Ventral anterior thalamus (vaTH)	5.70	−4.37	−2.45	9.13	0.46	0.42 ± 0.12
Contralateral hemisphere (right)						
Executive/insular						
Posterior insular cortex (pIC)	−9.50	−24.80	7.20	6.20	0.25	0.21 ± 0.05
Limbic/basal ganglia						
Caudate (CD)	−4.50	−17.87	−2.45	4.67	0.36	0.23 ± 0.09
Putamen (PU)	−6.10	−9.50	9.60	5.98	0.35	0.33 ± 0.05
Sensorimotor						
Premotor	−2.70	−19.70	16.80	5.05	0.34	0.31 ± 0.07

**Figure 4:**
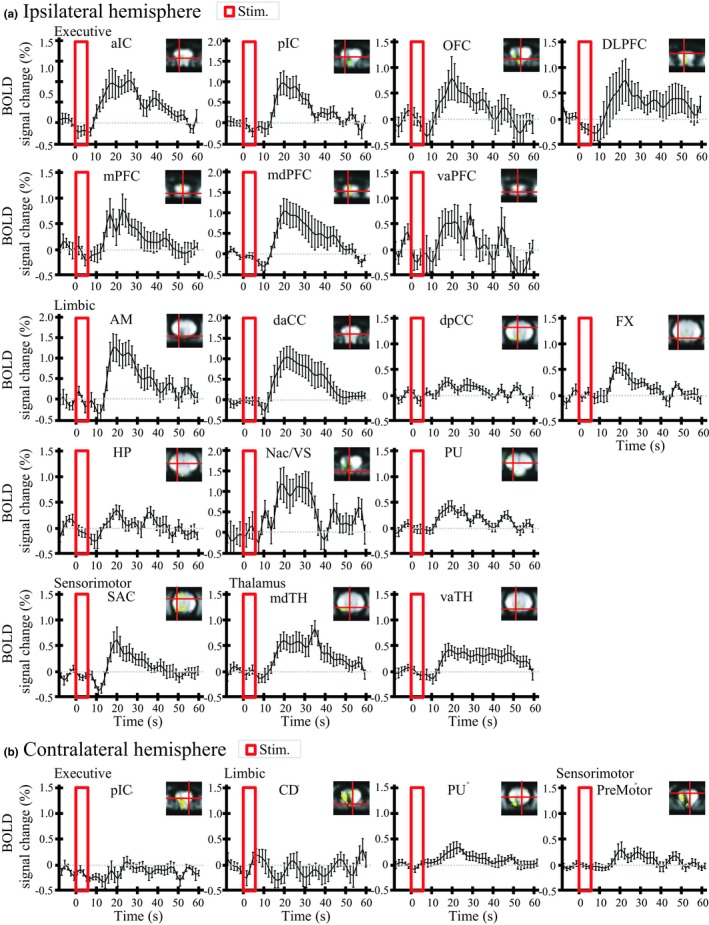
Time courses for the NAc‐DBS‐induced BOLD response in the ROIs (all subject averaged, *n* = 8) in (a) ipsilateral and (b) contralateral hemisphere. Six‐second stimulation period is denoted by red bars. The error bars indicate the ±1 standard error of the mean (*SEM*) of the BOLD signal. The location of each ROI is depicted in the upper right corner of individual figures. For the coordinates, see Table 1. NAc‐DBS evoked robust BOLD responses, characterized by an initial negative component followed by a peak (5 ± 3 s and 25 ± 5 s after stimulation onset, respectively). For abbreviations, see Appendix A

Figure 4 shows group‐averaged ROI time courses by individual region. The DBS evoked BOLD response was characterized by an initial negative induction (5 ± 3 s) followed by peaks (25 ± 5 s), which is consistent with previously published hemodynamic responses to visual and sensory stimulation (Ogawa et al., 1992, 1993).

### 3.2 Resting‐state functional connectivity

**Figure 5:**
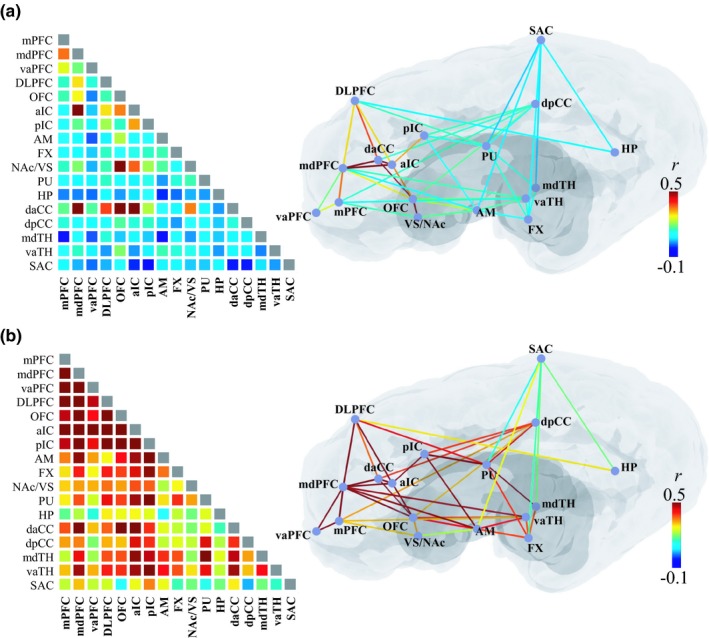
Pair‐wise functional connectivity (FC) is shown in the color encoded matrix (left) and illustrated with lines on a schematic brain diagram (right): (a) resting‐state functional connectivity (rsFC) and (b) stimulation‐state FC (“co‐activation”). Colors indicate the group‐averaged Pearson's correlation coefficient (*r*), ranging between −0.1 and 0.5. Significant resting or stimulation‐state functional connectivity (*p* < .05) is presented as colored lines on the brain illustration. Significant resting‐state correlations were found between NAc and multiple cortical and subcortical ROIs. During a stimulation period, multiple ROIs evoked highly significantly correlated BOLD signal activity at *p* < .05 (“co‐activation”). See for the abbreviations in Appendix A

In the resting‐state functional imaging results, mdPFC, daCC, aIC, pIC, OFC, and NAc showed significantly coherent inter‐regional BOLD activity (group‐average *r* = 0.21–0.65, *n* = 8, *t* > 2.37, *p* < .05, FDR corrected) (Figure 5a), indicating the presence of functional connectivity among those regions. In particular, NAc, of these ROIs, showed strong connectivity to the mdPFC, OFC, insula, and daCC, consistent with previously reported findings in animal (Morgane, Galler, & Mokler, 2005) and human studies (Cauda et al., 2011; Di Martino et al., 2008; Knutson, Adams, Fong, & Hommer, 2001). Additionally, daCC showed a broad connectivity to many other brain regions including the prefrontal regions, aIC, and NAc as shown in other resting‐state FC studies (Cauda et al., 2011; Greicius, Krasnow, Reiss, & Menon, 2003). Our results show that functional coupling could be observed between anatomically separate brain regions under conditions of isoflurane anesthesia.

### 3.3 Co‐activation of ROIs during stimulation

NAc‐DBS evoked temporally synchronized hemodynamic responses in ROIs of the ipsilateral prefrontal cortex (*r* > 0.3, *n* = 8, *t* > 2.37, *p* < .05) (Figure 5b). Importantly, it should be noted that co‐activation was found between regions, wherein their anatomical connections are less clear, such as those between the prefrontal cortex and the thalamus. These results support the fact that distal effect of NAc‐DBS cannot be fully explained by a direct inter‐regional anatomical connection alone, albeit the co‐activations in anatomically adjacent regions would not be surprising, that is, regions within limbic system or prefrontal cortex.

### 3.4 Relationship between resting‐state functional connectivity to NAc and amplitude of BOLD activation

**Figure 6:**
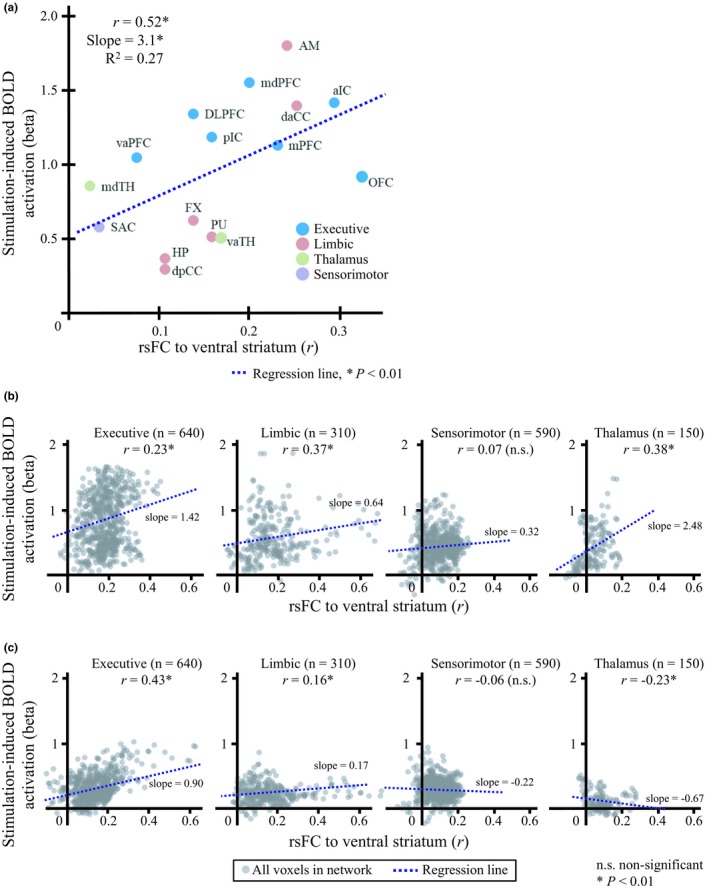
The relationship between resting‐state functional connectivity (rsFC) to the location of the stimulation (rsFC‐to‐NAc) and BOLD activation in (a) the ROIs, and all other voxels in (b) ipsilateral (c) and contralateral functional networks. The horizontal and vertical axes denote the rsFC (group‐averaged Pearson's correlation coefficient) of each ROI or voxel to NAc and the amplitude of BOLD activation (beta coefficient). The slope (*s*) of the linear regression is shown as a blue dotted line. Asterisks indicate a significant correlation between rsFC‐to‐NAc and BOLD activation in a given ROI or voxel of network. A significant linear relationship was found in the ROIs and voxels in ipsilateral functional networks. For the abbreviations, see Appendix A

We found that a significant positive relationship exists between the rsFC‐to‐NAc of individual ROIs and their BOLD response (*r* = 0.52, *p* < .01) (Table 1 and Figure 6a). The stronger rsFC to the stimulation locus (NAc) that was presented, the higher was the BOLD response. The relationship between the rsFC and BOLD response, however, was observed, not only in regions of activation above the statistical threshold (*p* < .05). Rather, the voxel‐wise analysis for expanded region of interests further revealed that BOLD response was correlated with the connectivity, that is, voxels in the bilateral executive and limbic networks (*r* = 0.16–0.43, *s* = 0.17–1.42, *p* < .01) (Figure 6b,c). Additionally, the relationship in the thalamic network was opposite from each other across the two hemispheres (*r* = 0.38 in the ipsilateral hemisphere, and *r* = −0.23 in the contralateral hemisphere) and no significant relationship was found in both sensorimotor networks. The results of both ROI and voxel‐wise analysis support the conclusion that functional connectivity, originated from the stimulation locus, may mediate the distal BOLD activation in remote regions.

### 3.5 The change in pair‐wise FC connectivity during poststimulation state

**Figure 7:**
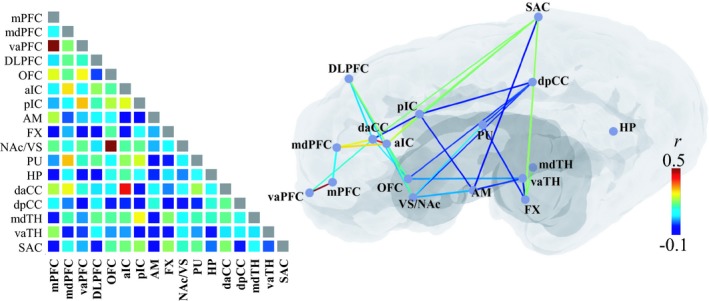
Pair‐wise functional connectivity (FC) during the poststimulation period presented in the color encoded matrix (left) and illustrated with lines on a schematic brain diagram (right). Colors indicate the group‐averaged Pearson's correlation coefficient (*r*), ranging between −0.1 and 0.5. A significant change in FC was found in subcortical ROI pairs (*p* < .05), shown as with colored lines on the brain illustration. NAc‐DBS suppresses functional connectivity between the ROIs and even alter the direction of connectivity from the positive to the negative in some ROI pairs. See for the abbreviations in Appendix A

**Figure 8:**
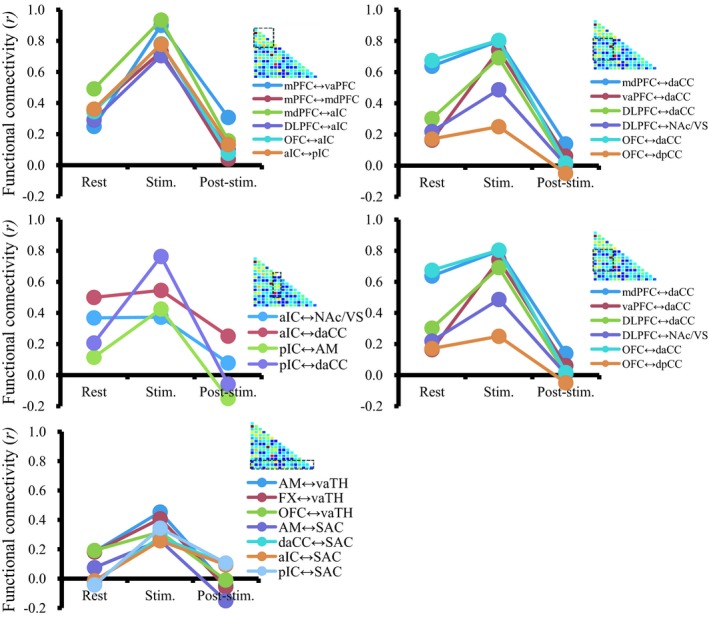
Significant changes in pair‐wise functional connectivity across resting, stimulation, and poststimulation states. The ROI pairs were grouped according to internetwork combinations for visualization (executive–executive, executive–limbic, insular–limbic, limbic–limbic, and limbic–others). The horizontal axis indicates three states, and the vertical axis indicates the functional connectivity (*r*). The poststimulation correlation coefficient matrix is shown on the upper right corner for reference. Pair‐wise FC is increased during the stimulation period (“co‐activation”) and decreased in poststimulation state (“inhibition”). See for the abbreviations in Appendix A

Significant decreases in FC (*p* < .05) were found between many regions in the poststimulation period, that is, daCC, dpCC, pIC, DLPFC, and vaTH (Figures 7 and 8); however, some others were enhanced after stimulation, that is, the pairs of VS‐mdPFC, mdPFC‐Pu, mdTH‐pIC, and mdTH‐OFC. Additionally, a few pair‐wise FCs became even negative after stimulation: pCC‐OFC, dpCC‐pIC, AM‐pIC, and AM‐SAC. The results indicate that NAc‐DBS, in general, have an inhibitory effect on major inter‐ROI networks after acute stimulation; however, the modulation effect of NAc‐DBS could also vary depending on the network being considered.

### 3.6 The relationship between poststimulation FC change and strength of resting‐state FC

**Figure 9:**
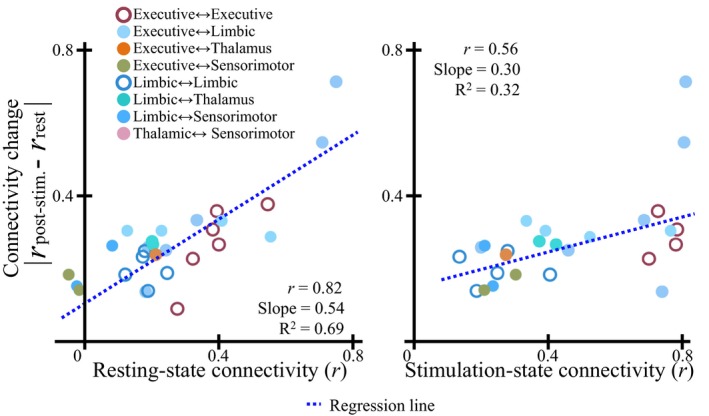
Relationship between inter‐ROI resting‐state functional connectivity and change in functional connectivity in the poststimulation state. Each circle represented an individual ROI pair, in which filled and unfilled circles differentiate the pairs between inter‐ and intranetwork. A significant, positive relationship was found between pair‐wise FC change and resting‐state FC (*r* = 0.82, slope = 0.54, *p* < .001) and stimulation‐state FC (*r* = 0.56, slope = 0.25, *p* < .01), respectively, demonstrating that resting and stimulation‐state connectivity could predict FC change in a given ROI pair

While it is not surprising that stimulation could alter the functional connectivity between ROIs, further results showed that the extent of FC change was correlated with the rsFC and the strength of co‐activation (Figure 9). A positively linear relationship was found between the change in FC in poststimulation and rsFC (*r* = 0.82, *p* < .05, *s* = 0.54), and stimulation‐state FC (“co‐activation”) in ROIs (*r* = 0.56, *p* < .05, *s* = 0.3), indicating that the effectiveness of stimulation‐induced modulation may vary depending on the rsFC.

### 3.7 Assessment of image artifacts induced by electric current

**Figure 10:**
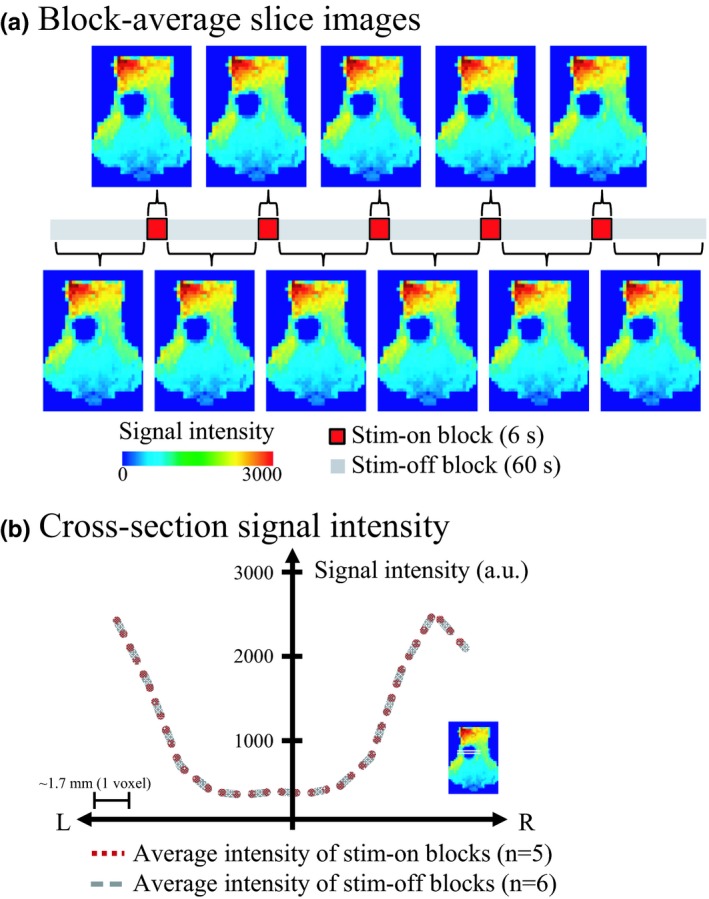
Assessment of an electric current‐induced EPI image artifact (a single subject). The intensity of the image signals was compared between electrical stimulation ON and OFF periods (a) block averaged axial image slices in stimulation ON and OFF block and (b) one dimensional plot of signal intensity across the “signal dropout region” for each condition. The horizontal axis denotes the distance from the center of signal dropout region, and the vertical axis denotes the voxel signal intensity. No significant difference in EPI images was found between stimulation ON and OFF periods, indicating that the influence of the electric current might be negligible

While it is known that the metal components of the DBS electrode can cause magnetic susceptibility artifacts as demonstrated by marked signal reductions in MR images near the implantation site (Settell et al., 2017), it is also possible that the electrical current of DBS could induce an additional image artifact, that is, increasing the size of the signal dropout area. The variation in image signal intensity was assessed near the signal dropout region during stimulation on and off periods; however, no statistically significant difference in signal intensity was found between stimulation on and off blocks (Figure 10). It therefore appears that the impact of electric current on the images is negligible.

## 4 DISCUSSION

NAc‐DBS‐induced BOLD activation occurs not only near the stimulation locus (NAc), but also in the distal regions across multiple functional networks that include ipsilateral prefrontal, limbic, thalamic, and sensorimotor areas. While the patterns and extent of BOLD activation were consistent with previous results in pig models and in human studies (Gibson et al., 2016; Knight et al., 2013; Rauch et al., 1994), previous studies have not addressed the mechanism involved in the propagating effect NAc‐DBS in the global brain. In our study, several ROIs lacking direct anatomical connections to the NAc showed temporally synchronized BOLD activity during stimulation (“co‐activation”), indicating that an anatomical connection alone cannot explain the diffuse pattern induced in a global brain. We therefore suggest that functional connectivity between stimulation locus and individual ROI and between ROIs likely play a crucial role in facilitating and propagating the DBS effect in distal brain regions.

### 4.1 Anatomy and functional connectivity of nucleus accumbens (NAc)

Our resting‐state analysis showed that NAc has significant resting‐state functional connectivity (rsFC) with the medial portion of the prefrontal cortex (OFC and mdPFC), cingulate cortex, insular cortex, and limbic substructures. These results indicate the presence of significant functional coupling between NAc and an executive network, which is not irrelevant to its anatomical position that links the prefrontal cortex and basal ganglia complex (Brog, Salyapongse, Deutch, & Zahm, 1993; Haber, Kim, Mailly, & Calzavara, 2006; Leh et al., 2007). NAc pathways play a key role in the development of reward‐guided behavior by associating reward information with motivational and emotional features of sensory inputs (Groenewegen, Wright, Beijer, & Voorn, 1999; O'Doherty, 2004; Reiss et al., 2005), that is, perceptual learning between visual stimuli and esthetic music reward enhanced the strength of FC between NAc and visual cortex (Salimpoor et al., 2013).

### 4.2 Local neuromodulatory effects of NAc‐DBS

Electrical stimulation directly modulates neuronal activity near the area of electrode implantation. Stimulation altered the spike rate of neurons and their firing pattern near stimulation locus (Anderson & Mullins, 2003; Dostrovsky & Lozano, 2002; Hashimoto, Elder, Okun, Patrick, & Vitek, 2003; McConnell, So, Hilliard, Lopomo, & Grill, 2012). Computation modeling simulated the extent of local effect (i.e., volume of tissue activated) (Butson & McIntyre, 2008; McIntyre & Grill, 1999). Our results also show that NAc‐DBS evoked the diffuse activation of BOLD at the outside of the implantation locus (Figure 3). Thus, the findings suggest that the electric field likely extends from the implantation locus to adjacent subcortical tissues of the NAc, presumably through the local neuronal network, although the extent may depend on the stimulation parameters being applied (e.g., voltage and frequency) (Paek et al., 2015; Settell et al., 2017).

### 4.3 Global neuromodulatory effects of NAc‐DBS

The modulation of electrical stimulation could be limited to a subset of afferent neurons (Canteras, Shammah‐Lagnado, Silva, & Ricardo, 1990); therefore, propagation of the effect to distal areas would be probabilistic and sparse in time (Chomiak & Hu, 2007; Hammond, Ammari, Bioulac, & Garcia, 2008). Such a limited local effect appears to somehow be in conflict with previous and present functional imaging results, wherein the DBS effect indeed extends to many distal regions. DBS‐fMRI studies have shown that DBS evokes changes in the cerebral metabolic rate and BOLD signal in broad cortical and subcortical areas (Asanuma et al., 2006; Fukuda et al., 2004; Haslinger et al., 2003; Jech et al., 2001; Le Jeune et al., 2010; Min et al., 2012; Rezai et al., 1999). Thus, these results suggest that a certain neurobiological mechanism is operative that allows the local modulatory effect to propagate to the global brain.

### 4.4 The role of functional connectivity in the propagation of NAc‐DBS effect

Some activated areas, on the one hand, appear to be anatomically linked to the NAc. For example, an anatomical connection has been identified between the NAc and prefrontal regions, as shown by DTI investigations (Britt et al., 2012; Brunenberg et al., 2012; Leh et al., 2007; Lehéricy et al., 2004). Some networks have been identified as involving the basal ganglia complex, that is, an “indirect” (Damoiseaux & Greicius, 2009; Montaron, Deniau, Menetrey, Glowinski, & Thierry, 1996), “hyper‐direct” pathway (Brunenberg et al., 2012; Nambu, Tokuno, & Takada, 2002), or the recurrent loops (Leblois, Boraud, Meissner, Bergman, & Hansel, 2006). On the other hand, however, the anatomical connection cannot fully explain the highly spread pattern of activation across large‐scale brain networks. Here, our results showed that the stronger the rsFC of a given region to the NAc was, the larger was the induced BOLD response (Figure 6). Thus, a functional connection that originated from the stimulation locus is highly likely to be the source of the BOLD activation pattern observed in the global brain. Furthermore, a greater FC change was observed in the poststimulation period when the rsFC in the two regions was stronger, supporting the idea that the rsFC may propagate the modulation effect in distal regions from the stimulation locus.

### 4.5 Functional connectivity change after NAc‐DBS

Many inter‐regional functional connectivity (FC) changes decrease after stimulation (Figure 7), indicating that the NAc‐DBS tends to have an inhibitory effect on functional connections. However, the magnitudes of reduction were not equal in the ROIs, and in some cases, the direction of functional coupling became reversed (i.e., negative correlation) or increased compared with the baseline condition (Figure 8). These results suggest that the DBS effect varies depending on the network being considered (Albaugh et al., 2016). It is generally thought that the DBS exerts its therapeutic effect by regularizing or normalizing a pathological oscillation in large‐scale networks (Chiken & Nambu, 2014; McIntyre & Hahn, 2010; Rosenbaum et al., 2014; Vitek, 2002). In line with the “network effect” perspective, we suggest that a variety of effects (inhibition or facilitation of functional connectivity) of DBS may reflect its regularizing efficacy for inter‐regional neuronal transmission, rather than simply inhibiting network activity.

The current steering technique with a novel lead design (Lehto et al., 2017; Martens et al., 2011) enables the shape of the electric field to be adjusted by the injection of an asymmetric current, thus providing better control of the DBS‐induced electric field (Butson & McIntyre, 2008). Functional imaging would then be better incorporated in terms of elucidating local and global modulation effects by adopting such selective stimulation to localize effects on the surrounding tissue of the NAc.

Note that we measured FC changes after a short period of stimulation (6 s) for a relatively short period of time (30 s) compared with the chronic DBS condition. Thus, the FC changes in the present study might reflect the acute effect of the NAc‐DBS. Further studies with a longer duration of stimulation would provide a more complete understanding of the effect of chronic DBS.

### 4.6 The influence of anesthesia on stimulation‐associated BOLD activity

The level of sedation (Liu, Zhu, Zhang, & Chen, 2013) and type of anesthetic agent (Angenstein, Kammerer, & Scheich, 2009; Angenstein, Krautwald, & Scheich, 2010) can have an influence on the BOLD hemodynamic response. Higher isoflurane concentrations (the dose over 1.8%) tend to suppress BOLD activity and results in a decrease in spatial specificity with different functional networks being dissolved during resting states (Liu et al., 2013). A burst suppression of the neuronal population was found in cases where high doses of isoflurane were used (Liu, Zhu, Zhang, & Chen, 2010; Vincent et al., 2007), suggesting that isoflurane as an anesthetic agent may have an impact on both hemodynamic and neuronal activity. In contrast to the high concentration, BOLD activation was preserved when a relatively lower dose (<1.3%) was used (Knight et al., 2013; Min et al., 2012; Paek et al., 2015), suggesting that the impact of isoflurane anesthesia would be less influential or negligible when a lower dose is used. It should be noted here that we cannot completely rule out the possibility that BOLD activation and the rsFC measurements were underestimated due to anesthesia. Nevertheless, we observed a robust amplitude (0.3%–1.3% signal change from the baseline) and temporal pattern of BOLD activation that are consistent with previous fMRI results (Knight et al., 2013; Min et al., 2012; Paek et al., 2015; Settell et al., 2017).

### 4.7 Clinical implications for human NAc‐DBS and Applications for DBS‐fMRI

Dysfunctional functional connectivity between the NAc and various brain regions has been implicated in a number of neuropsychiatric disorders (Da Cunha et al., 2015). Combining NAc‐DBS with functional imaging allows causal relationships between the stimulation and modulation of functional networks to be identified, thus providing insights into the underlying mechanisms responsible for the therapeutic and adverse effects of NAc‐DBS, particulary for neuropsychiatric disorders, for example, OCD and mood disorders (Bewernick et al., 2010; Bewernick, Kayser, Sturm, & Schlaepfer, 2012). Although our subjects were healthy animals, a large animal model can be implicated as a model for the human brain (Van Gompel et al., 2011), providing insights into how the modulation of these networks might underlie the potential therapeutic efficacy of human NAc‐DBS.

## CONFLICT OF INTEREST

The author(s) declare that there were no conflicts of interest with respect to the authorship or the publication of this article.

## AUTHOR CONTRIBUTIONS

All of the authors contributed to the study design. Data collection and analyses were performed by S. Cho, M. In, H. Min, and H. J. Jo. The manuscript was written by S. Cho, J. T. Hachmann, I. Balzekas, L. G. Andres‐Beck, and H. J. Jo in consultation with other authors. All the authors approved the final version of the manuscript for submission.

## Data Availability

The data that support the findings of this study are openly available in Data for: Resting‐state functional connectivity modulates the BOLD activation induced by nucleus accumbens stimulation in the swine brain at http://dx.doi.org/10.17632/r6f82ypfvt.3 (Cho, 2019).

## Appendix A

### Abbreviations

aIC, anterior portion of insular cortex

Am, Amygdala

CD, caudate

daCC, dorsal anterior cingulate cortex

DLPFC, dorsal lateral prefrontal cortex

dpCC, dorsal posterior cingulate cortex

FX, fornix

HP, hippocampus

mdPFC, mid‐dorsal lateral prefrontal cortex

mdTH, mediodorsal thalamic nucleus

mPFC, medial prefrontal cortex

NAc, nucleus accumbens

pIC, posterior portion of insular cortex

Pu, putamen

SAC, sensory association cortex

SNc, substantia nigra pars compacta

vaPFC, ventral anterior prefrontal cortex

vaTh, ventral anterior thalamic nucleus
